# Developmental Gene Expression Profiling along the Tonotopic Axis of the Mouse Cochlea

**DOI:** 10.1371/journal.pone.0040735

**Published:** 2012-07-12

**Authors:** Eun Jin Son, Ling Wu, Heejei Yoon, Sunhee Kim, Jae Young Choi, Jinwoong Bok

**Affiliations:** 1 Department of Otorhinolaryngology, Yonsei University College of Medicine, Seoul, Korea; 2 Department of Anatomy, Yonsei University College of Medicine, Seoul, Korea; 3 BK21 Project for Medical Science, Yonsei University College of Medicine, Seoul, Korea; Seattle Children’s Research Institute, United States of America

## Abstract

The mammalian cochlear duct is tonotopically organized such that the basal cochlea is tuned to high frequency sounds and the apical cochlea to low frequency sounds. In an effort to understand how this tonotopic organization is established, we searched for genes that are differentially expressed along the tonotopic axis during neonatal development. Cochlear tissues dissected from P0 and P8 mice were divided into three equal pieces, representing the base, middle and apex, and gene expression profiles were determined using the microarray technique. The gene expression profiles were grouped according to changes in expression levels along the tonotopic axis as well as changes during neonatal development. The classified groups were further analyzed by functional annotation clustering analysis to determine whether genes associated with specific biological function or processes are particularly enriched in each group. These analyses identified several candidate genes that may be involved in cochlear development and acquisition of tonotopy. We examined the expression domains for a few candidate genes in the developing mouse cochlea. *Tnc* (*tenacin C)* and *Nov (nephroblastoma overexpressed gene)* are expressed in the basilar membrane, with increased expression toward the apex, which may contribute to graded changes in the structure of the basilar membrane along the tonotopic axis. In addition, *Fst* (*Follistatin)*, an antagonist of TGF-β/BMP signaling, is expressed in the lesser epithelial ridge and at gradually higher levels towards the apex. The graded expression pattern of *Fst* is established at the time of cochlear specification and maintained throughout embryonic and postnatal development, suggesting its possible role in the organization of tonotopy. Our data will provide a good resource for investigating the developmental mechanisms of the mammalian cochlea including the acquisition of tonotopy.

## Introduction

The ability of the mammalian cochlea to respond to sounds of different frequencies is attributable to numerous specializations along the longitudinal (tonotopic) axis of the cochlea [1], [2]. Hair cells in the organ of Corti are the mechanosensory receptors that exhibit different sensitivities to specific frequencies depending on their position along the cochlear duct. Their morphological and mechanoelectrical properties gradually differ along the tonotopic axis, which appears to be critical for the frequency tuning [2]. Spiral ganglion neurons, which relay sound information from the hair cells in the form of electrical impulses to the brain, also display different morphological and electrophysiological characteristics along the length of the cochlea [1], [3]. Differences in synaptic structure, function, and number along the tonotopic axis have also been reported [4]. Besides the sensory receptors and neurons, nonsensory structures such as the basilar membrane and tectorial membrane also exhibit graded changes in composition and mechanical features. In fact, the basilar membrane, which vibrates in response to sound, has been considered as a primary determinant that stimulates the hair cells at a specific position with respect to a specific frequency [5]. The tectorial membrane, a viscoelastic structure that covers the stereociliary bundles of hair cells, has also been suggested to play a crucial role in facilitating tonotopic discrimination [6]. Despite numerous structural and cellular specializations in the cochlea that are thought to contribute to tonotopy, the molecular mechanisms that establish these graded specializations during development remain largely unknown.

Several studies have shown that genes and their isoforms are differentially expressed along the base and apex of the cochlea in both mammals and birds. Recently, the microarray technique has been utilized to obtain more comprehensive gene expression profiles along the tonotopic axis of the cochlea [7]–[9]. Sato and colleagues identified genes that are differentially expressed between basal and apical halves of the cochlea in adult mice [7]. In addition, several microarray analyses have compared gene expression levels along the tonotopic axis of the basilar papilla, the avian auditory organ, at hatching or 2-week-old chickens [8], [9]. These gene expression profiles will aid in understanding the tonotopic characteristics of the mature cochlea. However, since these analyses were performed after onset of hearing and the cochlea is thought to be mature already, they may not provide insight into how tonotopy is established.

In mice, the cochlea continues to develop after birth. The hair cells in the organ of Corti undergo dramatic architectural remodeling and achieve mechanosensitivity during the first week of postnatal development [10]–[12]. Also, the stereociliary bundles on the apical surface of the outer hair cells attain mature height and shape, leading to the characteristic organization of a stair-case pattern [10], [12]. The apical surface of the outer hair cells changes progressively from a rounded-hexagon to a non-convex circumference that correlates with the V-shape of their overlying stereociliary bundles [11]. These morphological changes are closely associated with redistribution of cytoskeletal proteins and spatiotemporal expression patterns of genes involved in mechanotransduction, which leads to maturation of the mechanosensitivity of the hair cells [10], [12], [13]. Moreover, the supporting cells in the organ of Corti also undergo major morphological and physiological changes during this period [14]. In particular, progenitor-associated genes such as Notch effectors are downregulated in supporting cells during postnatal development [15], [16]. The loss of Notch signaling may account for diminishment of stem cell features and lack of regeneration potential in the adult mouse cochlea [17], [18]. In contrast, genes associated with supporting cell maturation and function such as the intermediate filament glial fibrillary acidic protein (GFAP) and the glutamate transporter GLAST are upregulated during neonatal development [19], [20].

Interestingly, the morphological and functional maturation progresses gradually from the base of the cochlea toward the apex [10]–[12], [21]. Such changes precede the onset of auditory function, which suggests that the postnatal development may be crucial to achieving the tonotopic characteristics in the mammalian cochlea. Thus, we obtained comprehensive gene expression profiles along the tonotopic axis at postnatal day (P) 0 and P8, and compared expression profiles along the tonotopic axis (base, mid, apex) as well as between the neonatal stages (P0, P8). Our data should provide a platform for future investigations into the developmental mechanisms of the mammalian cochlea.

## Results and Discussion

### Hair Cell Maturation Along the Tonotopic Axis during Neonatal Cochlear Development

**Figure 1:**
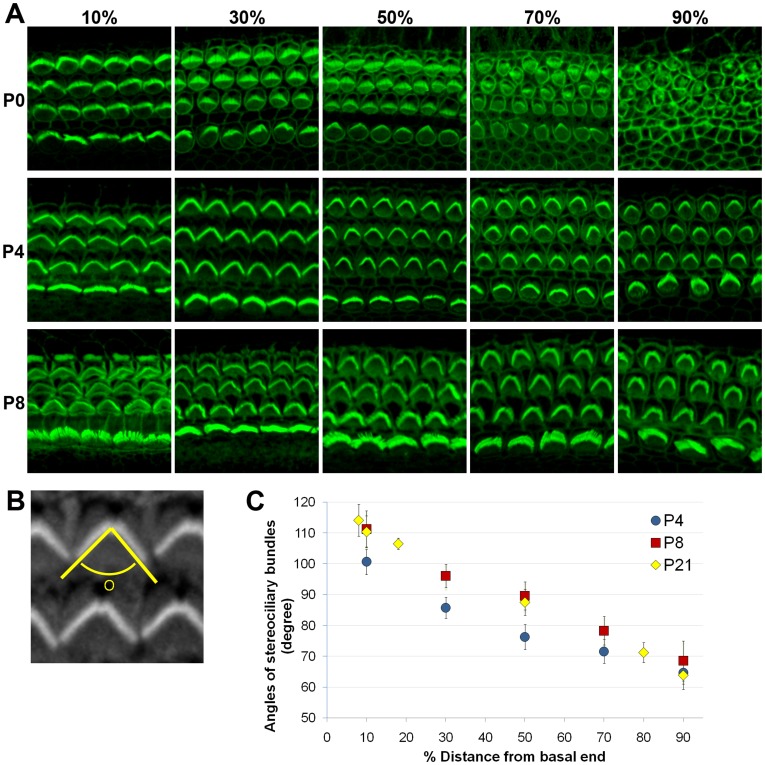
Maturation of hair cells during neonatal cochlear development. (A) Stereociliary bundles of the mouse cochlea were visualized by staining with Phalloidin at P0, P4 and P8. Pictures were taken from 10%, 30%, 50%, 70% and 90% positions from the basal end of the cochlear duct. (B) Angles of V-shaped hair bundles were measured by drawing two lines along the medial side of the bundles using the Image J program. (C) Angles of the hair bundles are plotted as a function of relative distance from the basal end of the cochlea at P4, P8, and P21.

It has been shown that hair cells in the mouse cochlea continue to differentiate after birth [10]–[12]. They acquire mature morphological and mechanoelectrical characteristics during the first postnatal week and hair cell differentiation progresses from the base of the cochlea towards the apex. We examined the maturation process of the hair cells during early postnatal stages in mice (Fig. 1). At P0, the basal half of the cochlea was organized into one row of inner and three rows of outer hair cells with distinct stereociliary hair bundles, while the apical region of the cochlea exhibited multiple rows of unorganized immature hair cells (Fig. 1A), At P4, the entire organ of Corti was in an orderly array with stereocilia of all outer hair cells displaying the characteristic V-shaped (Fig. 1A). The angle of the V-shaped hair bundles was wider at the base and smaller towards the apical cochlea (Fig. 1A). This gradual change in bundle angle is clearly shown in the graph plotted as a function of relative distance from the basal end (Fig. 1B&C). At P8, the morphologies of the hair bundles were almost identical with those of P4, but the angles were generally slightly wider and comparable to those of P21 (Fig. 1C). These results showed that hair cells in the developing cochlea gradually mature in a base-to-apex gradient, and the tonotopic variations along the cochlear duct become evident during neonatal development.

### Gene Expression Profiling Along the Tonotopic Axis during Neonatal Development

Our results and others suggest that neonatal development is crucial for maturation and acquisition of the tonotopic characteristics in the mouse cochlea (Fig. 1) [10], [11]. Genes that play important roles in these processes may be differentially expressed along the cochlear duct during neonatal stages. In order to obtain comprehensive gene expression profiles along the tonotopic axis of the mouse cochlea, we utilized the microarray technique. Approximately eighty temporal bones were dissected from P0 and P8 mouse pups and cochlear tissues containing the organ of Corti and lateral wall were collected. The cochlear tissues were divided into three equal pieces representing the base, middle and apex, and pooled separately (P0-base, P0-middle, P0-apex, P8-base, P8-middle, P8-apex). The same dissection procedure was repeated to obtain a second independent set of pooled cochlear samples from P0 and P8 mice. RNAs were purified from the two biological replicates and were independently subjected to microarray analyses.

The microarray data were analyzed to identify differentially expressed genes along the tonotopic axis at each stage (P0 or P8) by comparing the average expression levels of individual genes. Genes showing at least a 1.5-fold difference (*p*<0.05) between the base and the apex were considered to be differentially expressed. Interestingly, for most of the genes that were differentially expressed between the base and the apex, expression levels were gradually changed along the cochlear duct, exhibiting either an increasing or decreasing gradient towards the apex. Only a small fraction among the differentially expressed genes (<3%) showed peak expression in the middle cochlear region.

Table 1 shows the top fifty genes that showed higher expression in the base or apex at P0 or P8, ranked by the average fold differences (a complete list of the differentially expressed genes can found in Table S1). The lists contain many genes previously associated with inner ear development or function. For example, *Tectb, Isl1, Foxg1, Ush2a, Pcdh15,* and *Atoh1* showed higher expression in the apex at P0, while *Gjb6, Cldn11,* and *Otoa* showed higher expression in the base at P0. At P8, genes showing higher expression in the apex included *Tecta, Tectb, Tnc, Fgf10, Smpx, Slitrk6,* and *Otog,* while genes showing higher expression in the base included *Crym* and *Otos*. In addition, the lists contain many other genes that have not been previously associated with inner ear or hearing function.

**Table 1:** Genes differentially expressed along between apex and base of the mouse cochlea at P0 or P8.

P0 apex high		P0 base high		P8 apex high		P8 base high	
Gene	Fold diff. apex/base	Gene	Fold diff. apex/base	Gene	Fold diff. apex/base	Gene	Fold diff. apex/base
*Zpld1*	4.11	*Atp6v0a4*	0.24	*Tecta*	5.94	*Tnmd*	0.28
*Fst*	3.26	*Pkhd1l1*	0.28	*Tnc*	5.76	*Hoxa2*	0.28
*Ndst3*	2.97	*Hoxa2*	0.32	*Fst*	4.25	*Ifitm5*	0.35
*Clic6*	2.28	*Fcrlb*	0.33	*Fgf10*	3.55	*A2m*	0.40
*Sema3a*	2.21	*Ucma*	0.33	*Nov*	3.53	*Iyd*	0.41
*Tectb*	2.21	*Cyp3a13*	0.36	*Prkcq*	3.47	*Cntn6*	0.41
*Fam19a4*	2.17	*A2m*	0.37	*Isl1*	3.41	*Tmem27*	0.42
*Pcdh15*	2.16	*Vit*	0.40	*Chrna3*	3.28	*Tmem196*	0.42
*Zmat4*	2.16	*Slco1a4*	0.42	*Zpld1*	2.98	*Rarres1*	0.43
*Galntl6*	2.14	*Pkp1*	0.44	*Glb1l2*	2.93	*Trdn*	0.44
*Rorb*	2.10	*Gjb6*	0.47	*Syt1*	2.91	*Fam19a1*	0.44
*Fndc3c1*	2.07	*Kcna4*	0.47	*Smpx*	2.90	*Fam198b*	0.45
*Isl1*	2.03	*Cntn5*	0.48	*Gsbs*	2.81	*Crym*	0.46
*Tac1*	2.01	*Scg2*	0.48	*Slco5a1*	2.80	*Pi15*	0.47
*Slco5a1*	1.98	*Ppp1r1b*	0.50	*Muc15*	2.75	*Ptgfr*	0.47
*Epha3*	1.92	*Atp6v1c2*	0.51	*Plch1*	2.74	*Fgl2*	0.48
*Pcsk2*	1.85	*Clstn2*	0.51	*Ccdc148*	2.73	*Prss35*	0.49
*Hmga2*	1.82	*Spink5*	0.54	*Slitrk6*	2.72	*Pgf*	0.50
*Lrrc55*	1.80	*Wnk4*	0.54	*Fam40b*	2.70	*Dnase1*	0.50
*Tmem74*	1.80	*Cfh*	0.54	*Rbm24*	2.63	*H19*	0.51
*Trpa1*	1.79	*Rya3*	0.55	*Cybrd1*	2.60	*Rgs4*	0.51
*Tyrp1*	1.78	*Tcfcp2l1*	0.55	*Sox2*	2.56	*Myoc*	0.52
*Abcg5*	1.78	*Cldn11*	0.55	*Adamts19*	2.56	*Otos*	0.52
*P2rx3*	1.77	*Vcam1*	0.56	*Kcna10*	2.53	*Nox3*	0.52
*Pdzrn4*	1.75	*Mal*	0.56	*Itgb6*	2.50	*Ppp1r3c*	0.53
*Muc15*	1.73	*Anxa8*	0.56	*Rassf6*	2.45	*Smpd3*	0.53
*Cdh4*	1.72	*Akr1c14*	0.56	*Cubn*	2.39	*Ctgf*	0.53
*Fat3*	1.71	*Slc38a5*	0.57	*Otog*	2.38	*Agxt2l1*	0.54
*Angpt1*	1.70	*Oprk1*	0.57	*Slitrk3*	2.38	*Ntn1*	0.54
*Ush2a*	1.70	*Itga4*	0.57	*Pla2r1*	2.37	*Ppfia2*	0.54
*Slc39a8*	1.69	*Kcnj16*	0.57	*Slc26a5*	2.36	*Naip1*	0.54
*Dchs2*	1.68	*Adra2a*	0.58	*Epha3*	2.33	*Si*	0.55
*Rasef*	1.68	*Grin2a*	0.58	*Jag1*	2.32	*Gpr83*	0.55
*Csmd3*	1.67	*Lbp*	0.59	*Sfrp2*	2.32	*Slc38a4*	0.55
*Fam60a*	1.67	*Gabra5*	0.60	*Frmd3*	2.30	*Edil3*	0.55
*Foxg1*	1.66	*Car14*	0.60	*Xirp2*	2.29	*Pcolce2*	0.55
*Atoh1*	1.65	*Wee2*	0.62	*Il1rapl2*	2.26	*Kcna4*	0.55
*Robo2*	1.65	*Cited4*	0.63	*Spire2*	2.25	*Sema3d*	0.56
*Unc5c*	1.65	*Ndrg1*	0.63	*Robo2*	2.25	*Scn7a*	0.56
*Chrng*	1.64	*Adm*	0.64	*Tectb*	2.22	*Ly6a*	0.57
*Tmem2*	1.64	*Cntn1*	0.64	*Mybpc1*	2.22	*Matn4*	0.57
*Pls1*	1.64	*Agt*	0.64	*Kcnh8*	2.22	*Dio2*	0.57
*Mageb16*	1.63	*Aldh1l1*	0.64	*Rorb*	2.19	*Mup11*	0.57
*Plch1*	1.62	*Ano5*	0.64	*Pla2g4e*	2.19	*Pth1r*	0.57
*Prox1*	1.62	*Cmtm5*	0.64	*Prox1*	2.17	*Acan*	0.58
*Ar*	1.62	*Ampd3*	0.64	*Hs6st2*	2.14	*Fam180a*	0.58
*Prkg1*	1.61	*Tmem38b*	0.64	*Pcsk2*	2.13	*Mup7*	0.58
*Pappa2*	1.61	*Itgbl1*	0.65	*Tsga14*	2.12	*Ncam2*	0.58
*Scn9a*	1.61	*Otoa*	0.65	*Ush1c*	2.12	*Neto2*	0.58
*Six2*	1.59	*Slc17a7*	0.65	*Ppapdc1a*	2.11	*Atp13a5*	0.59

Lists of genes showing >1.5-fold difference (*p*<0.05) between apex and base at P0 or P8. Shown in the table are fifty genes for each group ranked by the average fold difference Fold difference was determined by comparing expression levels in two biological replicates, each of which were pooled from at least 80 cochlear tissues.

### Classification of Gene Profiles Based on Spatial and Temporal Expression Patterns

**Figure 2:**
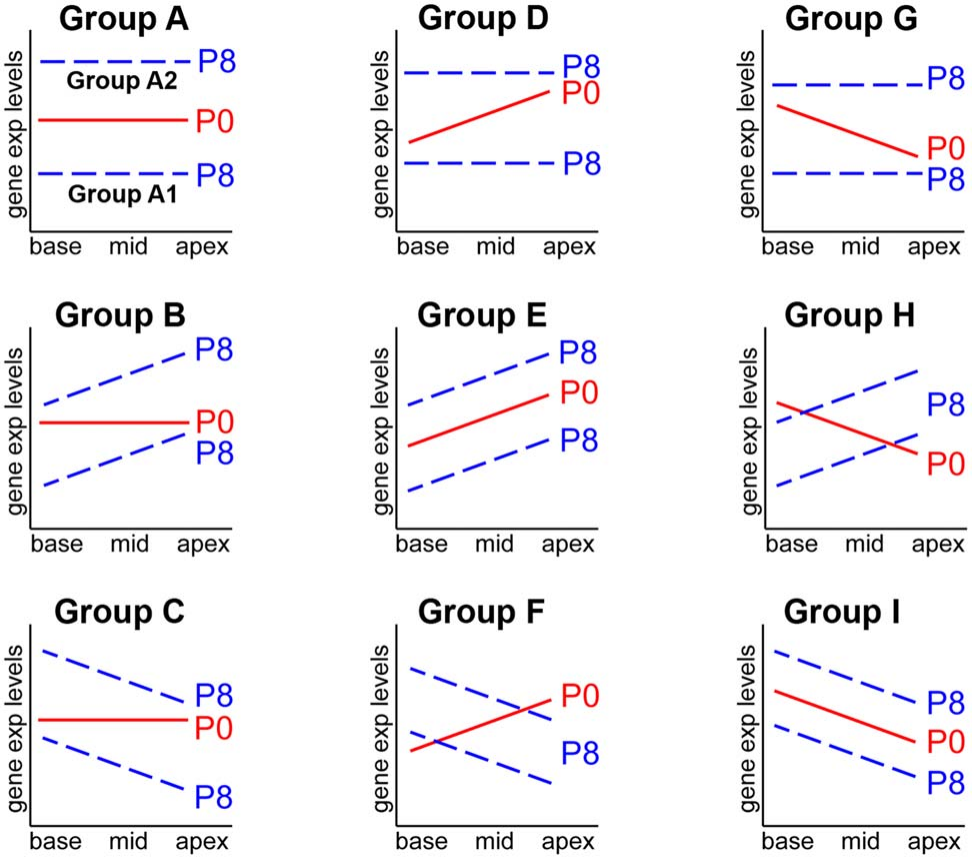
Schematic representation of distinct gene expression patterns along the tonotopic axis at P0 and P8. Gene profiles obtained from the microarray were classified based on the spatial (along the tonotopic axis) as well as temporal (between P0 and P8) expression changes. Expression levels of individual genes were compared between base and apex at P0, and classified into three categories: relatively constant along the cochlear duct (average fold-changes <1.5), higher in the apex or base (average fold-changes >1.5). Then, genes in each category were subdivided into three groups base on the expression patterns at P8 (constant, higher in the base or apex), which yielded nine distinct groups. See text for details.

We noticed that there were many genes that exhibited similar or different gradients at P0 and P8 (Table 1 and Table S1). For example, *Fst*, *Otog* and *Tectb* showed higher expression in the apex at both P0 and P8, but *Trpa1*, *P2rx3* and *Atoh1* were expressed more in the apex at P0, but no longer differentially expressed along the tonotopic axis at P8. Thus, we further classified the gene profiles according to the spatial (along the tonotopic axis) as well as temporal (between P0 and P8) expression patterns. The entire gene profiles were classified into three groups: 1) genes showing no gradient along the tonotopic axis, 2) genes showing increasing gradient towards the apex and 3) genes showing decreasing gradient towards the apex. Then, the expression patterns were compared between P0 and P8, which yielded nine groups displaying distinct expression patterns (Figure 2).

This analysis revealed that a majority of genes (>95%) were expressed relatively constantly along the tonotopic axis at both P0 and P8. Among these genes (no gradient at both P0 and P8), gene showing significant fold-changes between P0 and P8 (fold-change >2, *p*<0.05) were classified as Group A: some of them were down-regulated (Group A1) or up-regulated (Group A2) during this period. Among the genes showing no gradient at P0, some genes displayed an increasing (Group B) or decreasing (Group C) gradient at P8. Genes showing an increasing gradient toward the apex at P0 were also subcategorized into three groups, comprising genes no longer showing a gradient at P8 (Group D), retaining the increasing gradient (Group E) or showing the opposite (decreasing) gradient at P8 (Group F). Finally, genes showing a decreasing gradient toward the apex at P0 were similarly subcategorized as genes no longer showing a gradient at P8 (Group G), showing the opposite (increasing) gradient (Group H) or retaining the decreasing gradient at P8 (Group I). Examples of genes included in each group were shown in Table 2 (the complete list of the genes in each group can found in Table S2). The possible functional significance of the genes in each group is discussed below.

**Table 2:** Classification of genes differentially expressed along the tonotopic axis of the mouse cochlea during neonatal development.

			P0	P8				P0	P8				P0	P8
Group	Gene	P8/P0	apex/	apex/	Group	Gene	P8/P0	apex/	apex/	Group	Gene	P8/P0	apex/	apex/
			base	base				base	base				base	base
**A1**	*Gpr64*	0.16	1.42	1.25	**C**	*Tnmd*	3.95	0.86	0.28	**E**	*Fst*	0.72	3.26	4.25
	*Dcx*	0.18	1.27	1.44		*Ifitm5*	2.30	1.02	0.35		*Isl1*	0.50	2.03	3.41
	*Smoc2*	0.19	1.06	1.48		*Iyd*	1.92	0.90	0.41		*Zpld1*	0.82	4.11	2.98
	*Stmn2*	0.21	1.19	1.33		*Cntn6*	1.92	0.68	0.41		*Slco5a1*	0.50	1.98	2.80
	*Bex1*	0.25	1.25	0.72		*Rarres1*	4.54	1.27	0.43		*Muc15*	0.92	1.73	2.75
	*Agtr2*	0.25	0.80	0.89		*Trdn*	2.04	1.02	0.44		*Plch1*	1.02	1.62	2.74
	*Cldn6*	0.26	1.10	1.27		*Crym*	0.25	0.98	0.46		*Otog*	0.71	1.50	2.38
	*Bub1*	0.28	1.41	0.97		*Pi15*	1.22	0.76	0.47		*Pla2r1*	0.78	1.56	2.37
	*Spock3*	0.29	1.04	1.16		*Ptgfr*	1.79	0.87	0.47		*Epha3*	1.24	1.92	2.33
	*Igf2bp3*	0.29	1.19	0.93		*Fgl2*	1.75	1.11	0.48		*Jag1*	0.53	1.57	2.32
**A2**	*Clca5*	12.35	0.96	1.05		*Prss35*	0.80	1.20	0.49		*Robo2*	0.59	1.65	2.25
	*Slco1c1*	12.03	0.87	0.98		*Pgf*	0.99	0.91	0.50		*Tectb*	1.74	2.21	2.22
	*Phex*	9.13	1.02	0.75		*Mup2*	2.93	0.95	0.50		*Rorb*	4.03	2.10	2.19
	*Emilin2*	9.11	1.15	0.87		*Dnase1*	4.43	0.79	0.50		*Prox1*	0.39	1.62	2.17
	*Hhatl*	8.80	0.77	0.82		*H19*	0.58	1.08	0.51		*Pcsk2*	0.98	1.85	2.13
	*Scin*	8.79	1.12	1.08		*Rgs4*	2.73	0.98	0.51		*Ush1c*	1.10	1.52	2.12
	*Chad*	7.88	1.19	0.67		*Myoc*	3.67	1.09	0.52		*Chst15*	0.20	1.54	1.99
	*Ces1d*	6.82	1.45	1.47		*Otos*	6.17	0.76	0.52		*Ndst3*	0.56	2.97	1.78
	*Bhmt*	6.74	0.96	1.48		*Nox3*	1.06	0.85	0.52		*Fam70b*	0.57	1.53	1.75
	*Prss36*	5.93	1.02	0.73		*Smpd3*	4.97	1.17	0.53		*Clic6*	0.64	2.28	1.74
**B**	*Tecta*	0.22	1.32	5.94	**D**	*Zmat4*	0.38	2.16	1.18		*Foxg1*	0.47	1.66	1.71
	*Nov*	0.74	1.40	3.53		*Galntl6*	0.67	2.14	1.29		*Sema3a*	0.68	2.21	1.70
	*Chrna3*	4.01	0.89	3.28		*Fndc3c1*	0.43	2.07	0.97		*Igf2bp2*	0.72	1.57	1.68
	*Glb1l2*	1.18	1.10	2.93		*Tac1*	0.56	2.01	1.06	**F**	No gene is included			
	*Syt1*	0.61	1.27	2.91		*Trpa1*	0.53	1.79	1.15	**G**	*Atp6v0a4*	2.92	0.24	0.76
	*Smpx*	1.12	1.30	2.90		*Tyrp1*	0.84	1.78	0.91		*Pkhd1l1*	0.71	0.28	1.20
	*Gsbs*	2.63	1.08	2.81		*Abcg5*	0.43	1.78	1.17		*Ucma*	13.86	0.33	0.69
	*Ccdc148*	0.32	1.48	2.73		*P2rx3*	0.23	1.77	1.04		*Slco1a4*	8.81	0.42	0.75
	*Slitrk6*	0.83	1.13	2.72		*Pdzrn4*	0.84	1.75	1.10		*Pkp1*	1.94	0.44	0.71
	*Rbm24*	1.32	1.08	2.63		*Cdh4*	0.25	1.72	1.31		*Gjb6*	2.70	0.47	0.93
	*Cybrd1*	0.51	0.97	2.60		*Fat3*	0.56	1.71	1.01		*Cntn5*	0.23	0.48	0.79
	*Sox2*	0.51	1.33	2.56		*Angpt1*	0.63	1.70	1.35		*Scg2*	1.32	0.48	1.24
	*Adamts19*	0.51	1.47	2.56		*Slc39a8*	1.14	1.69	1.32		*Atp6v1c2*	2.09	0.51	1.05
	*Kcna10*	2.28	0.68	2.53		*Dchs2*	1.20	1.68	1.16		*Clstn2*	0.95	0.51	0.75
	*Rassf6*	1.98	1.29	2.45		*Csmd3*	0.54	1.67	1.02	**H**	*Fcrlb*	2.58	0.33	2.02
	*Cubn*	1.66	1.24	2.39		*Atoh1*	0.32	1.65	1.21	**I**	*Hoxa2*	1.16	0.32	0.28
	*Sfrp2*	0.43	1.14	2.32		*Unc5c*	0.79	1.65	0.84		*A2m*	2.87	0.37	0.40
	*Xirp2*	0.79	0.98	2.29		*Chrng*	0.69	1.64	1.12		*Kcna4*	1.81	0.47	0.55
	*Il1rapl2*	1.73	1.25	2.26		*Pls1*	0.54	1.64	1.48		*Grin2a*	0.76	0.58	0.65
	*Spire2*	0.94	1.36	2.25		*Mageb16*	0.78	1.63	1.13		*Ppp1r1b*	2.17	0.50	0.66

Genes showing >1.5-fold difference (*p*<0.05) between apex and/or base (apex/base) were included in the classification. Fold difference between P0 and P8 was calculated with the average values of base, middle, and apex in each age. Shown in the table are examples of genes included in each group. A complete list can be found in the Table S2.

### Validation of the Microarray Data with Quantitative RT-PCR

**Figure 3:**
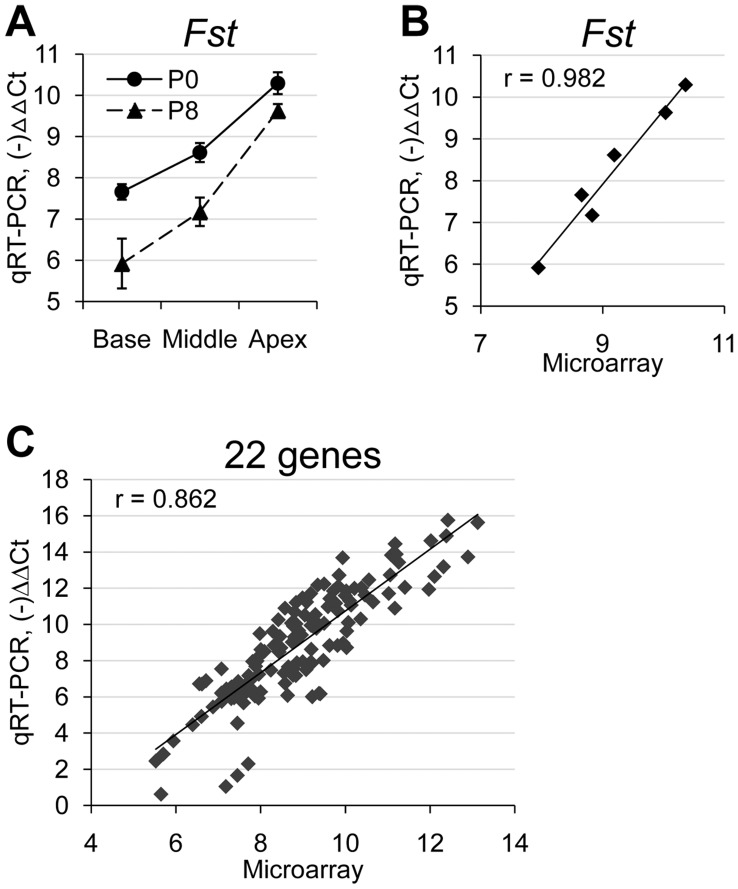
Comparison between qRT-PCR and microarray data showed high correlation. (A) An example of qRT-PCR results for *Fst*, which showed increasing base-to-apex gradients in expression levels at both P0 and P8. (B) An example showing high correlation between qRT-PCR results and microarray data for *Fst* (Pearson correlation coefficient, r = 0.982). (C) Comparisons between qRT-PCR results and microarray data for all 22 genes examined showed good correlations (r = 0.862).

We validated our microarray data by performing quantitative real time PCR (qRT-PCR) on 22 genes selected from various groups (Fig. 3 and Table 3). Genes for qRT-PCR were selected among ones showing higher fold changes in each group such as *Stmn2*, *Tecta*, *Tnmd*, *Fst*, *Atp6v0a4*, *Hoxa2*, and *A2m*, and also among ones previously shown to be associated with cochlear development or function such as *Kcnj10*, *Kcnq1*, *Ptprq, Tectb, Otog* and *Ush1c*. As an example, qRT-PCR results for *Fst* (*Follistatin*: Group E) showed an increasing gradient toward the apex at both P0 and P8 (Fig. 3A). When the qRT-PCR results were plotted as a function of the values from the microarray, close correlation was clearly shown (Fig. 3B, Pearson correlation coefficient, *r* = 0.982). Similarly, all 22 selected genes generally showed close correlations between the microarray data and qRT-PCR results (Pearson correlation coefficient, *r* = 0.862) (Fig. 3C).

**Table 3:** Validation of microarray data with quantitative RT-PCR.

		Average fold change (Apex/Base)					Average fold change (P8/P0)^2^	
		Microarray		qRT-PCR		Pearson’s Correlation Coefficient	Microarray	qRT-PCR
Group	Gene	P0(A/B)	P8(A/B)	P0(A/B)	P8(A/B)		(P8/P0)	(P8/P0)
**A1**	*Stmn2*	1.19	1.33	2.38	2.84	0.949	0.21	0.08
	*Ccnb1*	1.37	0.90	1.64	0.89	0.995	0.34	0.24
**A2**	*kcnj10*	0.69	0.90	0.42	0.88	0.999	3.71	21.88
	*Kcnq1*	0.82	0.85	0.56	0.52	0.960	2.13	3.17
**B**	*Tecta*	1.32	5.94	2.20	7.56	0.957	0.22	0.03
	*Ptprq*	0.78	2.11	0.27	2.87	0.837	1.17	1.81
	*Chrna10*	0.84	1.92	0.55	2.96	0.796	1.11	0.83
	*Fzd4*	1.15	1.54	1.74	1.09	0.900	1.39	1.69
	*Nrcam*	1.40	1.99	1.84	2.55	0.989	0.62	0.51
**C**	*Tnmd*	0.86	0.28	0.81	0.12	0.981	3.95	17.79
	*Crym*	0.98	0.46	1.48	0.24	0.982	0.25	0.12
**D**	*Pdzrn4*	1.75	1.10	2.80	1.15	0.845	0.84	0.70
	*Cdh4*	1.72	1.31	2.89	1.08	0.997	0.25	0.04
**E**	*Fst*	3.26	4.25	6.23	13.07	0.982	0.72	0.41
	*Tectb*	2.21	2.22	4.86	12.86	0.953	1.74	2.87
	*Otog*	1.50	2.38	1.48	3.84	0.982	0.71	0.59
	*Ush1c*	1.52	2.12	1.75	3.93	0.967	1.10	0.98
**G**	*Atp6v0a4*	0.24	0.76	0.06	0.54	0.996	2.92	10.59
	*Slco1a4*	0.42	0.75	0.41	0.36	0.992	8.81	13.70
**I**	*Hoxa2*	0.32	0.28	0.06	0.01	0.942	1.16	0.85
	*A2m*	0.37	0.40	0.21	0.16	0.997	2.87	3.95
	*Ppp1r1b*	0.50	0.66	0.26	0.35	0.969	2.17	4.95

2 Fold changes between P0 and P8 (P8/P0) were calculated using the average values of the base, middle, and apex for each age.

In Table 3, we calculated fold changes between the apex and base with the results from qRT-PCR, and compared to those from the microarray. For genes from Group A showing no gradient along the tonotopic axis, average expression levels between P0 and P8 were compared. The results from qRT-PCR appeared to be more sensitive than the microarray data in verifying the changes between the base and the apex. Nevertheless, the expression patterns from the qRT-PCR results were generally consistent with those from the microarray.

### Functional Annotation Clustering Analysis

Our microarray analysis yielded an extensive list of genes that are differentially expressed along the tonotopic axis during neonatal development. Analysis of the dynamic expression patterns of individual genes may highlight some key players that are critical to cochlear maturation including the acquisition of tonotopy (Table 2 and Table S2). However, these single-gene analyses may fail to identify important biological processes, which are often regulated collectively by a group of genes involved in common biological function or pathways. Also, biologically significant gene expression changes occurring in a certain group of cells can be missed due to masking by genes expressed in other cell types or inherent noise signals. Thus, in order to evaluate our microarray data at the level of biological function and processes, we performed functional annotation clustering analysis using the Database for Annotation, Visualization, and Integrated Discovery (DAVID) bioinformatics resources [22]. This analysis identified biological functions and processes specifically enriched in the gene list of each group, which were summarized in Table 4.

**Table 4:** Functional annotation clustering analysis on the differentially expressed genes.

Group	Total genes	Enrich. Score^1^	Biological annotations	Ratio (%)^2^	p-value^3^	FDR (%)	Genes
**A1**	168	8.42	GO:0022402∼cell cycle process	13.13	1.15E-10	1.75E-07	*Bub1 C79407 Ccna2 Ccnb1 Ccnb2 Cdca3 Cks2 Dbc1 Dbf4 Dlgap5 f630043a04rik Hells Kif11 Mki67 Ncaph Plk1 Racgap1 Sgol2 Spag5 Stmn1 Tpx2*
		6.86	GO:0005694∼chromosome	15.0	1.24E-13	1.51E-10	*Bub1 C79407 Cenpk F630043a04rik H2afy2 H2afz Hells Hist1h1a Hist1h1b Hist1h2ab Hist1h2af Hist1h2an Hist1h2ao Hist1h2bb Hist1h2bj Hist1h2bl Hist1h2bm Hist1h2bn Hist1h3a Hist1h3b Hist1h3c Hist1h3d Hist1h3e Hist1h3g Hist1h3h Hist1h3i Hist2h3b Hist2h3c1 Hm11277 Hmgb2 Hmgb3 Mki67 Sgol2 Spag5 Tmpo Top2a Zfp57*
		1.92	GO:0031012∼extracellular matrix	6.9	3.61E-04	0.43868	*Aspn Col6a3 Egfl6 Fbn2 Fras1 Matn1 Oc90 Postn Smoc2 Spock3 Tgfbi*
		1.68	GO:0022832∼voltage-gated channel activity	4.4	0.00254	3.18051	*Cacna1h Kcna6 Kcnh7 Scn2a1 Scn3a Scn3b Scn9a*
		1.51	GO:0015630∼microtubule cytoskeleton	10.0	7.94E-06	0.00968	*Ccnb1 Ckap2 Dlgap5 F630043a04rik Kif2c Kif4 Kif11 Kif15 Kif21b Kif23 Myc Racgap1 Sncg Spag5 Stmn1 Tpx2*
**A2**	245	8.94	GO:0005578∼proteinaceous extracellular matrix	9.2	4.31E-10	5.3E-07	*Abi3bp Chad Chl1 Col4a3 Col4a4 Col8a1 Col24a1 Cpz Ecm2 Emilin2 Entpd2 Fgf1 Gpld1 Kazald1 Kera Lama2 Lgals3bp Npnt Ntn4 Prss36 Spon1 Tnfrsf11b Wnt2b*
		5.25	GO:0007155∼cell adhesion	8.8	1.56E-05	0.02553	*Anxa9 Atp1b2 Bcam Cd9 Cd36 Cd44 Chl1 Clca5 Cntn4 Col4a3 Col8a1 Col24a1 Emilin2 Itga10 Lama2 Lgals3bp Ninj2 Npnt Sorbs3 Spon1 Tesk2 Wisp1*
		1.78	GO:0006811∼ion transport	8.4	0.00113	1.82672	*Atp1b2 Atp1b3 Atp2a3 Cacng4 Clca5 Fxyd3 Fxyd5 Glrb Kcne4 Kcnj2 Kcnj10 Kcnj13 Kcnq1 P2rx2 Slc5a5 Slc9a2 Slc10a4 Slc41a2 Slco1c1 Steap3 Tcn2*
**B**	125	2.41	GO:0030182∼neuron differentiation	0.8	0.0015	2.28299	*Gata2 Isl2 Lingo1 Mtap2 Nr2e1 Nrcam Slitrk2 Slitrk6 Sox2 Wnt7a*
		1.88	GO:0044459∼plasma membrane part	2.4	4.47E-05	0.05379	*Apba1 Aqp4 Aqp5 Chrna3 Chrna7 Chrna10 Chrnb4 Cldn9 Cldn23Cubn Cybrd1 Egflam Faim2 Fn1 Kcna10 Kcnb2 Nrcam OtoF Otog Pclo Pla2g4e RgS6 Rgs9 Syt1 Rims2 Six1 Slc6a14 Slc22a3 Sox2 Tecta Tmprss9 Xirp2*
**C**	84	2.12	GO:0005578∼proteinaceous extracellular matrix	0.9	0.00373	3.92815	*Acan Crtap Ctgf Nid2 Ntn1 Trf Vcan*
		1.74	GO:0006812∼cation transport	1.2	7.95E-04	1.13346	*Atp13a5 Kcnh5 Kcnma1 Scn7a Slc4a10 Slc5a3 Slc38a4 Slc39a14 Trf*
**D**	46	1.87	GO:0033555∼multicellular organismal response to stress	7.4	7.93E-05	0.11043	*P2rx3 Scn9a Tac1 Trpa1*
**E**	27	3.01	GO:0030182∼neuron differentiation	28.6	9.95E-07	0.00138	*Foxg1 Isl1 Jag1 Rorb Robo2 Sema3a Ush1c Ush2a*
		1.77	GO:0007423∼sensory organ development	25.0	1.34E-06	0.00186	*Foxg1 Jag1 Prox1 Rorb Six2 Ush1c Ush2a*
		1.53	glycosylation site:N-linked (GlcNAc…)	46.4	0.00117	1.44099	*Ndst3 Fst Jag1 Epha3 Pcsk2 Zpld1 Robo2 Chst15 Sema3a Tectb Pla2r1 Muc15 Ush2a*
**F**	0		No gene set was significantly enriched				
**G**	42	1.94	GO:0006811∼ion transport	21.3	7.43E-05	0.10516	*Ano5 Atp6v0a4 Atp6v1c2 Cp Gabra5 Kcnj16 Slc17a7 Slco1a4 Tmem38b Wnk4*
		1.66	GO:0007155∼cell adhesion	17.0	5.79E-04	0.81684	*Agt Cldn11 Clstn2 Cntn1 Cntn5 Itga4 Pkp1 Vcam1*
**H**	1		No gene set was significantly enriched				
**I**	5		No gene set was significantly enriched				

1 Enrichment scores indicate relative importance (enrichment) of the biological annotations.

2 Ratio (%) indicates the percentage of the genes included the enriched annotations among the total number of genes in the group.

3 *p*-value was calculated with a modified Fisher’s exact test to indicate the significance of the enrichment of the annotations.

In Group A1 (no gradient, down-regulated at P8), genes associated with cell cycle process or chromosomal activity were enriched. Such enrichments indicated that cell proliferation occurring at P0 is generally diminished at P8, which is consistent with active maturation processes observed during neonatal cochlear development. Interestingly, genes associated with microtubule cytoskeleton such as kinesin family members were also shown to be down-regulated at P8, which may be related with a reduced cell cycle and with disassembly of the microtubule-based kinocilium in the hair cells during this period.

On the other hand, Group A2 (no gradient, up-regulated at P8) contained many genes associated with extracellular matrix and cell adhesion such as *Col4a3, Col4a4 and Emilin2*. Upregulation of these genes may explain the maturation of the cytoarchitecture in the cochlea during the postnatal period. Consistent with our results, *Emilin2* was shown to be rapidly accumulated during postnatal development in the extracellular matrix surrounding the collagenous fibers in the basilar membrane [23]. Genes associated with ion transport such as *Kcnq1, Kcne1* and *Kcnj10* were also enriched. It has been shown that *Kcnq1* and *Kcne1* are expressed in strial marginal cells and that *Kcnj10* in intermediate cells plays a critical role in potassium recycling in the endolymph [24]. Thus, enrichment of such gene sets in P8 cochlea is consistent with the establishment of endocochlear potential during the first postnatal week in the mouse cochlea [25].

In Group B (no gradient at P0, increasing base-to-apex gradient at P8), genes associated with neuron differentiation and plasma membrane were enriched. Several genes involved in cochlear development and function were included in this group such as *Tecta*, *Gata2*, *Slitrk6*, *Chrna10, Nrcam, Otof* and *Otog*. The higher expression of these genes in the apex at P8 may be related to the base-to-apex wave of cochlear maturation, since the increasing base-to-apex gradient for some genes resulted from down-regulation at the base by P8. In support of this notion, two genes in this group, *Ptprq* and *Tecta*, which are both important for inner ear development, were no longer expressed in the adult cochlea [26], [27]. Interestingly, several genes related to non-sensory structures such as the basilar or tectorial membrane were included in this group. *Tecta* and *Otog*, which encode for α-tectorin and otogelin, respectively, that are components of the tectorial membrane [28], were expressed at higher levels in the apex. *Tectb*, encoding β-tectorin and classified in Group E, also showed higher expression in the apex at P8. The higher expression levels of genes associated with the tectorial membrane in the apex may be related with the decreasing base-to-apex gradient in stiffness of the tectorial membrane, an important factor for tonotopic discrimination [29].

In Group C (no gradient at P0, decreasing base-to-apex gradient at P8), genes related to extracellular matrix were enriched. The biological annotation for proteinaceous extracellular matrix was also shown to be enriched in Group A2 with different sets of genes, suggesting that different types of extracellular matrix components are required at different stages of postnatal cochlear development. Also enriched were genes associated with cation transport. Interestingly, splicing variants of *Kcnma1*, which encodes for the Maxi-K^+^ (BK) channel have been shown to be differentially expressed along the tonotopic axis and also during developmental stages in mice and chicken [8], [30]. *Kcnma1* expression patterns from our microarray data, showing no gradient at P0 and higher expression in the base at P8, did not closely match with previous reports. This may be because the microarray technique did not distinguish the splicing variants, but displayed a summed expression level of all the variants expressed at the time of analysis. Since differential expressions of specific splice variants have been suggested to contribute to tonotopy [2], it may be necessary to profile the entire transcriptome along the tonotopic axis using deep-sequencing technologies such as the RNA-seq approach [31].

In Group D (increasing base-to-apex gradient at P0, no gradient at P8), enriched genes were related with multicellular organismal responses to stress including *P2rx3* and *Trpa1*. Consistent with our data, *P2rx3,* encoding the P2X_3_ subunit of ATP-gated ion channels, was shown to be expressed at higher levels in the apical cochlea at P0, and its expression is gradually decreased and no longer detected after the onset of hearing [32]. Similarly, expression of *Trpa1*, a subunit for transient receptor potential (TRP) channels, was greatly increased in the apex at around P0 and then decreased to show no gradient by P6 [13]. These expression changes precede the onset of hearing, suggesting their roles in the acquisition of mature cochlear function such as the mechanosensitivity of the hair cells.

In Group E (increasing base-to-apex gradient at both P0 and P8), genes associated with neuron differentiation and sensory organ development were enriched, which is consistent with the ongoing maturation processes in the apical cochlea during neonatal stages (Fig. 1) [10], [11].

In Group G (decreasing base-to-apex gradient at P0, no gradient at P8), genes related to ion transport and cell adhesion were enriched. For most genes in this group, the expression gradient at P0, higher in the base, was lost at P8 due to increased expression of these genes towards the apex during the postnatal development. Since ion transport and cell adhesion molecules are closely associated with cochlear remodeling and function, this expression pattern may be related with the base-to-apex wave of cochlear maturation. Consistently, these gene sets were also shown to be enriched in Group A2, in which genes are up-regulated at P8, showing no gradient.

Finally, in Groups F (higher expression in the apex at P0 and in the base at P8), H (higher expression in the base at P0 and in the apex at P8) and I (higher expression in the base at both P0 and P8), no functionally related genes were significantly enriched.

### Confirmation of Expression Patterns for Selected Genes by in Situ Hybridization

We next performed in situ hybridization for selected genes to confirm their differential expression patterns along the tonotopic axis. We selected a few genes previously shown to be expressed in the cochlea such as *Chrna10*, *Ptprq*, *Kcnj10*, *Tectb*, and *Tnc*, as well as a few genes that exhibited higher fold changes along the tonotopic axis among the newly identified genes from our analysis such as *A2m*, *Nov*, and *Fst*.

We first performed in situ hybridization on *Chrna10*, *Ptprq*, *Kcnj10*, and *Tetcb*, whose expression patterns have previously been reported, to confirm our microarray results [26], [27], [33], [34]. Expression domains of these genes within the neonatal cochlea were consistent with the previous reports, and their graded expression patterns along the tonotopic axis generally matched with the microarray data (see Fig. S1 and Fig. S2 for details). Expression pattern of *A2m* was also consistent with the microarray data (Fig. S2).

**Figure 4:**
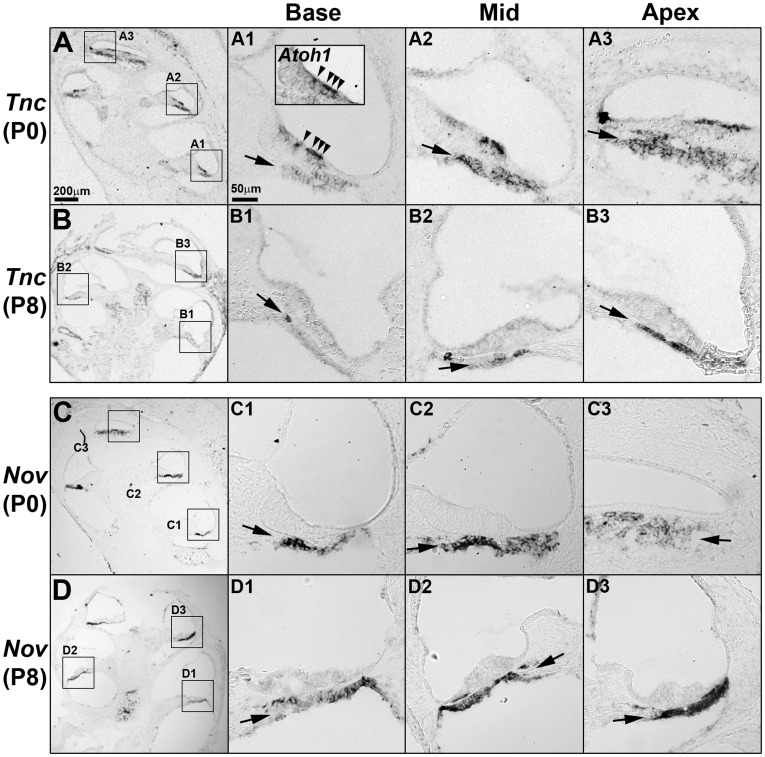
Expression patterns of *Tnc* and *Nov* in the cochlea during neonatal development. Expression patterns of *Tnc* (A,B) and *Nov* (C,D) were examined by in situ hybridization at P0 (A,C) and P8 (B,D). (A,B) *Tnc* transcripts were observed in the basilar membrane in an increasing gradient toward the apex at both P0 and P8 (A1–B3, arrows). *Tnc* expression was also positive in the differentiating hair cells (*Atoh1* expression domain) at P0 (inset in A1, arrowheads), but was down-regulated at P8. (C,D) *Nov* was expressed in the basilar membrane at P0 and P8. *Nov* expression levels were relatively constant at P0 along the cochlear duct and gradually decreased toward the apex by P8 (D). Scale bar in A (200 µm) applies to B, C, and D; scale bar in A1 (50 µm) applies to A1–A3, B1–B3, C1–C3, and D1–D3.

We next examined the expression patterns of the few newly identified genes from our microarray analysis. We found that *Nov* (*nephroblastoma overexpressed gene*), identified in Group B, was expressed in the basilar membrane with no obvious gradient at P0 and in an increasing base-to-apex gradient at P8, consistent with the microarray results (Fig. 4C,D, arrows). The *Nov* (also called as *CCN3*) gene encodes for a matricellular protein that associates with the extracellular matrix and mediates a variety of cellular functions including proliferation, differentiation, survival, migration and cytoskeleton reorganization [35]. Since the structural organization of the extracellular matrix regulates the mechanical properties of the basilar membrane, gradual changes in *Nov* expression may contribute to the frequency tuning of the basilar membrane [5]. The expression pattern of *Nov* in the basilar membrane was compared with that of *Tnc*, which encodes the glycoprotein tenascin C and has been shown to be expressed in the developing mouse cochlea including the basilar membrane [36]. *Tnc* was strongly expressed in the hair cells and the basilar membrane along the cochlear duct at P0 (Fig. 4A,B). While *Tnc* expression in the hair cells was downregulated at P8 (Fig. 4A,B, arrowheads), its expression in the basilar membrane continued in an increasing base-to-apex gradient at P8, consistent with the microarray results (Table 1). The specific expression in the basilar membrane with gradual changes along the cochlear duct of *Nov* and *Tnc* suggest that these extracellular matrix proteins may be involved in the developmental regulation of the basilar membrane, specifying differential properties along the tonotopic axis.

**Figure 5:**
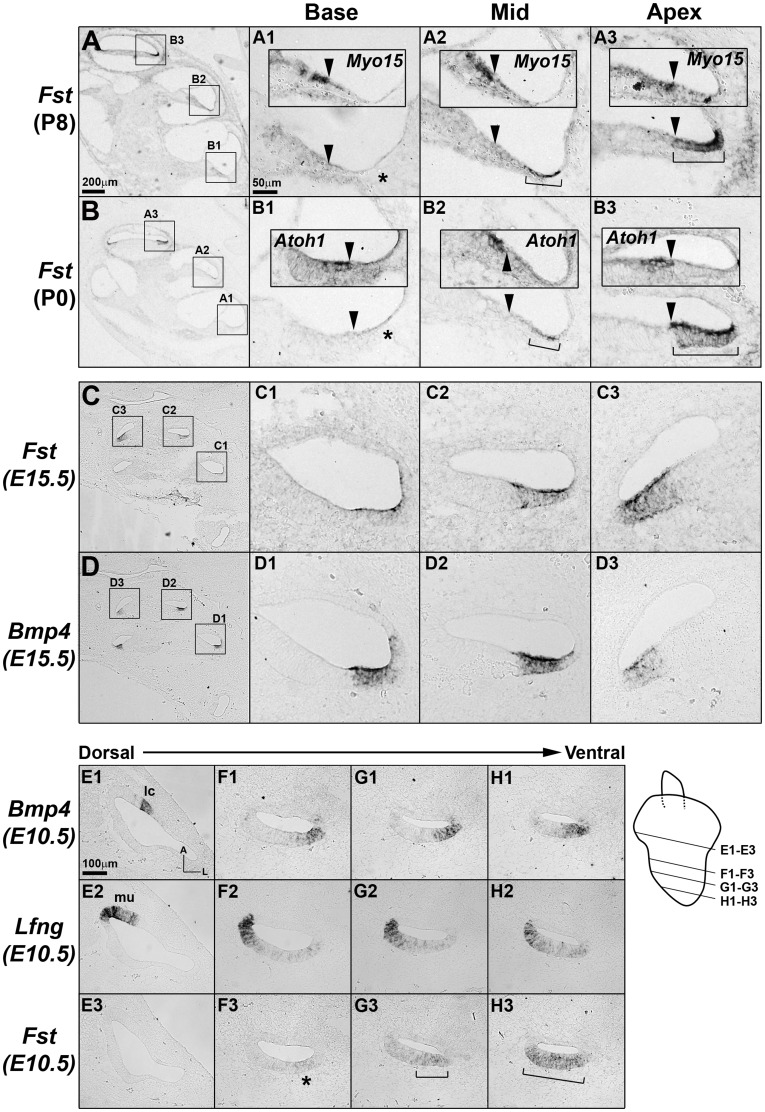
Expression patterns of *Fst* in the cochlea during embryonic and neonatal development. *Fst* transcripts were present in the lesser epithelial ridge (LER) region of the cochlea in an increasing base-to-apex gradient during neonatal (A,B) and embryonic development (C-H). (A,B) At P0 and P8, *Fst* expression was barely detectable in the basal cochlear turns (A1,B1, asterisks), but was evident in the lateral side of the LER in the middle turns (A2,B2, brackets) and its expression domain included the entire LER region in the apical turns (A3,B3, brackets). Insets in A1–B3 show expression domains of *Myo15* or *Atoh1* in the adjacent sections to indicate the location of hair cells. Arrowheads indicate the lateral border of differentiating hair cells. (C,D) *Fst* expression patterns at E15.5 were comparable to those observed at P0 and P8. *Fst* expression domains largely overlapped that of *Bmp4* in the LER area. (E1–H3) The increasing gradient of *Fst* expression patterns in the cochlea was already apparent in the cochlear primordium at E10.5 (F3,G3,H3, asterisk, brackets). *Fst* expression was not detectable in the presumptive vestibular organs including the lateral crista (E1–E3). Expression domains of *Bmp4* (E1,F1,G1,H1) and *Lfng* (E2,F2,G2,H2) indicated the lateral and medial regions of the developing cochlea, respectively. The schematic diagram indicates the level of sections for the pictures shown in E1–H3. Scale bar in A (200 µm) applies to B-D; scale bar in A1 (50 µm) applies to A1–A3, B1–B3, C1–C3, and D1–D3; Scale bar in E1 (100 µm) applies to E1–H3.

We also examined the expression patterns of *Fst* (*Follistatin*), a TGF-β/BMP antagonist, which was identified in Group E and displayed high fold-changes along the tonotopic axis at both P0 and P8 (Table 1 and 2). *Fst* expression was barely detected in the basal turn of the cochlear duct, began to be observed in the lateral side of the lesser epithelial ridge (LER) in the middle turn and then expanded to the entire LER in the apical turn at both P0 and P8 (Fig. 5A,B, brackets). Since *Bmp4* and *Bmp7* are also expressed in the LER region (Fig. 5D) [37], the gradual expansion of the *Fst* expression domain implies gradual decreases of BMP activities toward the apex. We then asked when this graded *Fst* expression pattern is established. At E15.5 when cochlear morphogenesis is almost complete, the graded expression patterns of *Fst* were comparable to those observed at P0 and P8 (Fig. 5C). Thus, we examined the inner ears at E10.5, when the primordial cochlear structure just begins to form in the ventral aspect of the otocyst. When examined from dorsal to ventral sections of the otocyst, *Fst* expression was not detected in the vestibular structures (Fig. 5E1–E3), and began to be faintly observed at the level where *Bmp4* and *Lfng* were expressed in the lateral and medial side of the developing cochlea, respectively (Fig. 5F1–F3) [38]. *Fst* expression became stronger and broader toward the apical cochlear sections, while *Bmp4* and *Lfng* expression patterns were not changed (Fig. 5G1–H3, brackets). However, at E9.5 when the cochlear anlage has yet to be developed, *Fst* expression was not detected in the otocyst (data not shown). These results indicate that the longitudinal gradient of *Fst* expression is established at the time of cochlear specification and maintained throughout cochlear development including embryonic and neonatal stages. Considering the role of BMP signaling in hair cell differentiation and inner ear development [37], [39], [40], the graded expression patterns of *Fst* suggest a possible role for Follistatin in the specification of gradually different properties along the cochlear duct such as the tonotopy.

### Conclusions

In this study, we applied the microarray technique to identify genes that are differentially expressed along the longitudinal axis of the mouse cochlea during neonatal development. Our analyses identified several candidate genes, which may be involved in cochlear development and acquisition of tonotopy, but have not been appreciated previously. Although the biological relevance of these candidates in cochlear development remains to be determined, the gene expression profiles obtained in this study will be a useful resource for studying various aspects of mammalian cochlear development.

## Materials and Methods

### Tissue Dissection and RNA Extraction

All animals were handled in accordance with the guidelines for the Care and Use of Laboratory Animals of Yonsei University College of Medicine. The protocol for obtaining cochlear samples was approved by the Committee on Animal Research at Yonsei University College of Medicine (#10-104). Eighty temporal bones were dissected from C57BL/6 mice at P0 and P8. After removing the otic capsule, the cochlear tissues including the organ of Corti, spiral limbus, and lateral wall was dissected in sterile, chilled Hanks’ balanced salt solution under a stereomicroscope (Olympus SZX10). The cochlear tissues were divided into three equal pieces representing the base, middle and apex, and pooled separately (P0-base, P0-middle, P0-apex, P8-base, P8-middle, P8-apex). The pooled cochlear tissues were homogenized with a micro-homogenizer (Microtec, Chiba, Japan), and total RNA was extracted using an RNeasy Mini Kit (Qiagen, Hilden, Germany). The quality and quantity of the extracted total RNA was measured using the Total RNA Nano Assay (Agilent Technologies, Palo Alto, CA, USA). The same procedure was repeated to obtain additional biological replicates for each cochlear sample (P0-base, P0-middle, P0-apex, P8-base, P8-middle, P8-apex).

### Microarray Data Analysis

Gene expression profiles were generated using the Affymetrix GeneChip© Mouse Gene 1.0 ST oligonucleotide arrays (Affymetrix, Santa Clara, California). RNA samples isolated from two biological replicates were sent to the local Affymetrix GeneChip© service provider (DNA LINK, Seoul, Korea). Three hundred nano-grams of high quality total RNA was converted into double-stranded cDNA using a random hexamer conjugated with a T7 promoter sequence. Amplified RNA (cRNA) was generated from the double-stranded cDNA using an in vitro transcription (IVT) reaction and then re-converted into cDNA with a mix of dNTPs containing dUTP. This cDNA was then fragmented by uracil-DNA glycosylase (UDG) and APE1 restriction endonucleases and labeled with a biotinylated dideoxynucleotide via a terminal transferase reaction. End-labeled cDNA was hybridized for 16 hours at 45° and 60 rpm according to the Gene Chip Whole Transcript (WT) Sense Target Labeling Assay Manual (Affymetrix). After hybridization, the chips were stained and washed in a Genechip Fluidics Station 450 (Affymetrix) and scanned using a Genechip Array scanner 3000 7G (Affymetrix). Affymetrix Command Console Software version 1.1 was used to process the raw images into CEL files. Gene expression estimates were normalized using the robust multiarray averaging (RMA) method. Microarray data were deposited in the GEO database (#GSE34187) and followed MIAME requirements.

Genes showing an average fold-change greater than 1.5 (*p*<0.05, unpaired t-test) between the base and the apex and exhibiting a log-transformed gene expression level greater than 6 were considered to be significantly differentially expressed. The differentially expressed genes were further classified into nine groups based on the fold-changes along the tonotopic axis (base vs. apex) and during neonatal development (P0 vs. P8) (Fig. 2). Gene lists in each group were subjected to functional annotation clustering analysis using the Database for Annotation, Visualization, and Integrated Discovery (DAVID) (http://david.abcc.ncifcrf.gov/home.jsp). The analysis was performed by following the procedure previously described [22]. Biological annotations with an enrichment score of >1.5, P-value (<0.01) and false discovery rate (FDR, ≤5%) were considered biologically interesting (Table 4).

### Quantitative Real-time PCR

Quantitative real-time PCR (qRT-PCR) was performed to confirm the microarray data. RNAs purified from three biological replicates for each of the cochlear samples (two biological replicates used for the microarray analysis and one additional (third) biological replicate collected on a small scale) were used for qRT-PCR. cDNAs were synthesized from each RNA (2 µg) sample using poly (dT)_20_ and the Superscript III RT kit according to manufacturer’s instructions (Invitrogen). No-RT controls were not included. cDNAs were diluted three times with water and the same amount of cDNAs were used for all PCR reactions. qRT-PCR for 22 selected genes was performed in triplicate wells using SYBR Green PCR Master Mix and the ABI 7500 machine (Applied Biosystems, Carlsbad, California). The samples were denatured at 94°C for 10 minutes and then subjected to 40 cycles of denaturing at 94°C for 15 seconds and annealing and extension at 60°C for 1 minute. We normalized gene expression level (ΔCt) with β-actin (*Actb*). Relative expression levels were determined by comparative methods (ΔΔCt). Relationships between the microarray data and qRT-PCR were assessed with Pearson correlation coefficient (r) (Table 3). Primers used for qRT-PCR were designed by using the Primer3 program [41]. At least one primer (either forward or reverse) was designed to include an exon-exon junction sequences to avoid amplification of genomic DNA. All the primer sequences are listed in Table S3.

### Phalloidin Staining and Measurement for the Angles of Stereociliary Bundles

Stereociliary bundles were visualized by staining the organ of Corti with Alexa Fluor® 488 Phalloidin (Invitrogen) at P0, P4, P8 and P21. Pictures were taken from 10%, 30%, 50%, 70%, and 90% positions from the basal end of the cochlea duct using a confocal microscopy (Zeiss LSM700). The angle formed by two lines drawn along the medial side of the hair bundles of outer hair cells was measured using the Image J program (National Institutes of Health, USA) (Fig. 1B). Angles for at least thirty outer hair cells were measured for each cochlear region and plotted as a function of relative distance from the basal end of the cochlea.

### In Situ Hybridization

In situ hybridization was performed as previously described [38]. Probes for *Atoh1* [42], *Myo15* [43], *Bmp4* and *Lfng* [38] were prepared as previously described. RNA probes for *Fst* were generated from a 313 base pair (bp) mouse *Fst* cDNA containing the +36 to +348 coding region (MN_008046); for *Tnc*, from a 404 bp mouse *Tnc* cDNA containing the +5835 to +6060 coding region and 178 bp 3′ untranslated region (NM_011607); for *Nov*, from a 966 bp mouse *Nov* cDNA containing the 38 bp 5' untranslated region and +1 to +928 coding region (NM_010930); for *Chrna10*, from a 645 bp mouse *Chrna10* cDNA containing the +882 to +1344 coding region and 182 bp 3′ untranslated region (NM_001081424); for *Ptprq*, from a 455 bp mouse *Ptprq* cDNA containing the 2 bp 5' untranslated region and +1 to +453 coding region (NM_001081432); for *Kcnj10*, from a 714 bp mouse *Kcnj10* cDNA containing the +749 to +1140 coding region and 322 bp 3′ untranslated region (NM_001039484); for *Tectb*, from a 406 bp mouse *Tectb* cDNA containing the +801 to +990 coding region and 216 bp 3′ untranslated region (NM_009348); for *A2m*, from a 1018 bp mouse *A2m* cDNA containing the 180 bp 5' untranslated region and +1 to +1000 coding region (NM_175628).

## Supporting Information

Figure S1 **Expression patterns of** ***Tectb, Chrna10,*** **and** ***Ptprq*** **in the cochlea during neonatal development.** Expression patterns of *Tectb* (A,B), *Chrna1* (C,D) and *Ptprq* (E, F) were examined by in situ hybridization at P0 (A,C,E) and P8 (B,D,F). (A,B) *Tectb* was classified in Group E showing increasing gradients towards the apex at both P0 and P8. Consistent with the microarray data, *Tectb* showed higher expression towards the apex at both P0 (A1–A3) and P8 (B1–B3). In the middle turn of P0 cochlea, *Tectb* expression was found in the lateral margin of greater epithelial ridge, pillar cells, and the third row of Deiter’s cells, consistent with a previous report [26]. Expression domains of *Tectb* were either diminished in the base or expanded in the apex (A1–A3). At P8, *tectb* expression in the pillar cells was disappeared and its increasing gradient towards the apex maintained (B1–B3). (C, D) *Chrna10* was classified in Group B showing no gradient at P0 and an increasing gradient towards the apex at P8. Consistently, *Chrna10* is expressed in the differentiating hair cells relatively constantly along the tonotopic axis at P0 (C1–C3) and in an increasing gradient towards the apex at P8 (D1–D3). (E, F) *Ptprq* was also classified in Group B showing no gradient at P0 and an increasing gradient towards the apex at P8. However, *Ptprq* transcripts are observed in the differentiating hair cells with slightly higher expression in the base at P0 (E1–E3, arrowheads), which represents the qRT-PCR results more rather than the microarray data (Table 3). At P8, *Ptprq* transcripts are observed in an increasing gradient towards the apex at P8, consistent with the microarray and qRT-PCR results (F1–F3). Both *Ptprq* and *Chrna10* were down-regulated in the base at P8, which may reflect the base-to-apex progression of hair cell maturation.
(TIF)

Figure S2 **Expression patterns of** ***A2m*** **and** ***Kcnj10*** **in the cochlea during embryonic and neonatal development.** Expression patterns of *A2m* (A,B) and *Kcnj10* (C,D) were examined by in situ hybridization at P0 (A,C) and P8 (B,D). (A,B) *A2m* was classified in Group I showing decreasing gradients towards the apex at both P0 and P8. Consistent with the microarray data, *A2m* transcripts are observed in the Reissner’s membrane in a decreasing gradient toward the apex at P0 (A1–A3). Interestingly, *A2m* expression is no longer detected in the Reissner’s membrane at P8, but is observed in the spiral ligament area retaining the decreasing gradient toward the apex (B1–B3). *Kcnj10* was classified in Group A1 showing no gradient at both P0 and P8 with up-regulation at P8. Consistent with the microarray data, *Kcnj10* expression was barely detected in the P0 cochlea, but clearly observed in the stria vascularis and the organ of Corti at P8.
(TIF)

Table S1 **Complete list of the differentially expressed genes between apex and base of the mouse cochlear at P0 or P8.**
(XLSX)

Table S2 **Complete list of genes in each group (Groups A to H), classified based on spatial and temporal expression patterns.**
(XLSX)

Table S3 **Primer sets used for qRT-PCR.**
(DOCX)
