# Supplementary figures and images for: Developmental Gene Expression Profiling along the Tonotopic Axis of the Mouse Cochlea
Source: PLoS One. 2012 Jul 12;7(7):e40735. doi: 10.1371/journal.pone.0040735 (PMC3395647; doi:10.1371/journal.pone.0040735)

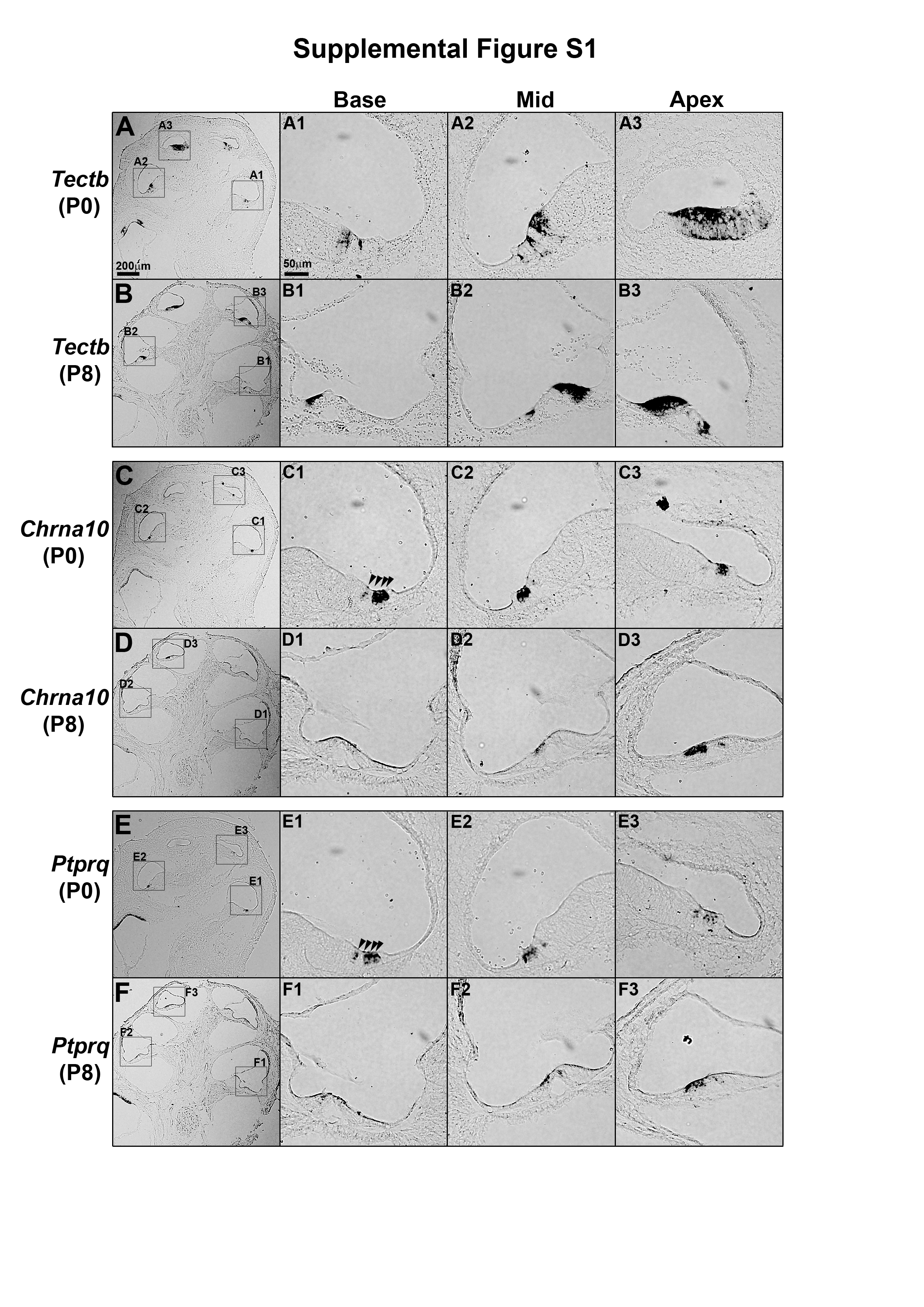

Supplement: Figure S1 — Expression patterns of Tectb, Chrna10, and Ptprq in the cochlea during neonatal development. Expression patterns of Tectb (A,B), Chrna1 (C,D) and Ptprq (E, F) were examined by in situ hybridization at P0 (A,C,E) and P8 (B,D,F). (A,B) Tectb was classified in Group E showing increasing gradients towards the apex at both P0 and P8. Consistent with the microarray data, Tectb showed higher expression towards the apex at both P0 (A1–A3) and P8 (B1–B3). In the middle turn of P0 cochlea, Tectb expression was found in the lateral margin of greater epithelial ridge, pillar cells, and the third row of Deiter’s cells, consistent with a previous report [26]. Expression domains of Tectb were either diminished in the base or expanded in the apex (A1–A3). At P8, tectb expression in the pillar cells was disappeared and its increasing gradient towards the apex maintained (B1–B3). (C, D) Chrna10 was classified in Group B showing no gradient at P0 and an increasing gradient towards the apex at P8. Consistently, Chrna10 is expressed in the differentiating hair cells relatively constantly along the tonotopic axis at P0 (C1–C3) and in an increasing gradient towards the apex at P8 (D1–D3). (E, F) Ptprq was also classified in Group B showing no gradient at P0 and an increasing gradient towards the apex at P8. However, Ptprq transcripts are observed in the differentiating hair cells with slightly higher expression in the base at P0 (E1–E3, arrowheads), which represents the qRT-PCR results more rather than the microarray data (Table 3). At P8, Ptprq transcripts are observed in an increasing gradient towards the apex at P8, consistent with the microarray and qRT-PCR results (F1–F3). Both Ptprq and Chrna10 were down-regulated in the base at P8, which may reflect the base-to-apex progression of hair cell maturation. (TIF) [file pone.0040735.s001.tif]

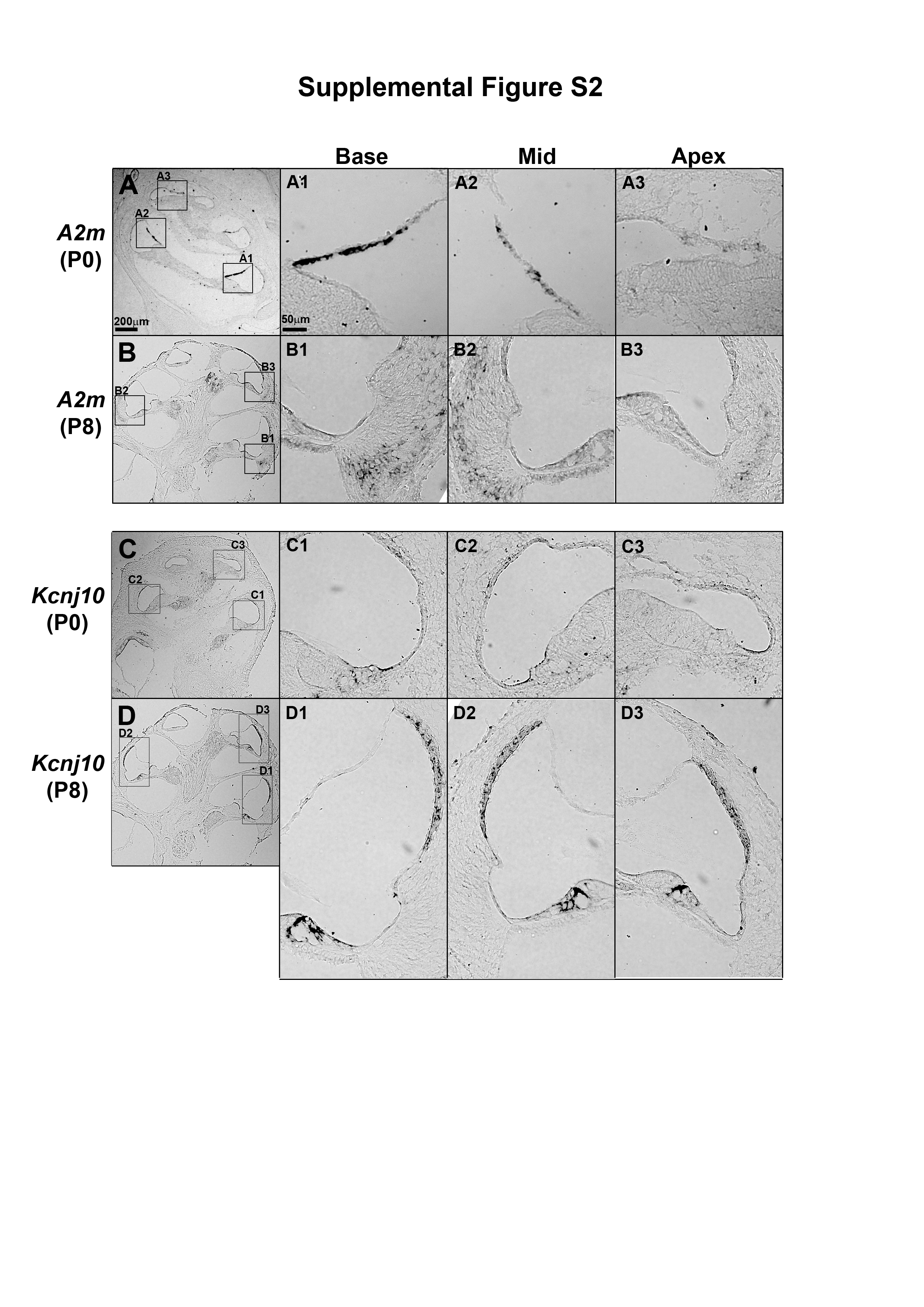

Supplement: Figure S2 — Expression patterns of A2m and Kcnj10 in the cochlea during embryonic and neonatal development. Expression patterns of A2m (A,B) and Kcnj10 (C,D) were examined by in situ hybridization at P0 (A,C) and P8 (B,D). (A,B) A2m was classified in Group I showing decreasing gradients towards the apex at both P0 and P8. Consistent with the microarray data, A2m transcripts are observed in the Reissner’s membrane in a decreasing gradient toward the apex at P0 (A1–A3). Interestingly, A2m expression is no longer detected in the Reissner’s membrane at P8, but is observed in the spiral ligament area retaining the decreasing gradient toward the apex (B1–B3). Kcnj10 was classified in Group A1 showing no gradient at both P0 and P8 with up-regulation at P8. Consistent with the microarray data, Kcnj10 expression was barely detected in the P0 cochlea, but clearly observed in the stria vascularis and the organ of Corti at P8. (TIF) [file pone.0040735.s002.tif]
