# Supplementary material for: Developmental Gene Expression Profiling along the Tonotopic Axis of the Mouse Cochlea
Source: PLoS One. 2012 Jul 12;7(7):e40735. doi: 10.1371/journal.pone.0040735 (PMC3395647; doi:10.1371/journal.pone.0040735)
Supplement: Table S3 — Primer sets used for qRT-PCR. (DOCX) [file pone.0040735.s005.docx]

Supplemental Table S3. Primer sets used for qRT-PCR

| **Gene** | **Forward primer** | **Reverse primer** |
| --- | --- | --- |
| *Actb* | CTTCTTGGGTATGGAATCCTG | TCAGGAGGAGCAATGATCTTG |
| *Stmn2* | CATCTACACCTACGACGACATG | TTTTCTGAATCTCCTCCAGAGAC |
| *Tnmd* | CCCACTCTAATAGCAGTTTCAG | AGGTCTTCCTCGCTTGCTTG |
| *Crym* | TTCTGTTGTCAGGGGCTGAC | TGGCTGCAACCAGGTCTTC |
| *Pdzrn4* | CCCGACATGCAGCTGGATG | TTCTCCTGGTTGTTGGAAGAG |
| *Cdh4* | GAGACTTCATTAATGAGGGACTC | CCCAGTCATTTAAGTAGTCGTAATC |
| *Slco1a4* | ATAAATAGCTTCAGGCGCATTTAC | ACTTGCTTTCCTTCGCAGTG |
| *Hoxa2* | TGCCTCGGCCACAAAGAATC | CTCTCGGTCAAATCCAGCAG |
| *A2m* | GGTGTGTCTACCTCCAGACATC | GAACGGCTTCCAATGTAACTG |
| *Ppp1r1b* | CCATCACTGAAAGCTGTGCAG | TTCTTCTTCGTCCTCCTCTTC |
| *Ccnb1* | CCTCACAAAGCACATGACTGTC | ATGGTGCCAACTGCATCTG |
| *Kcnj10* | CACCTTCGAGCCAAGATGAC | CAGCAATGTGCTCCATTCTC |
| *Kcnq1* | AGGGTGGAAGACAAGGTGAC | CACAGTCAGTTGTTCGTAGGTG |
| *Chrna10* | CTCTTTCTCCCTGCAGAGTG | CAGGTACAAGGTCAGCACTTG |
| *Ptprq* | TTGAGAAAGGACGGATCAGATG | ATGCAGTCTCCATGCCTTTC |
| *Fzd4* | CAGCCAGCTGCAGTTCTTC | ACATGTGGTTGTGGTCGTTC |
| *Tecta* | TTAGG AGAAAAAGGC TGGACTG | CACTGAAGAATTAGAGGCTCTG |
| *Nrcam* | GACGTTTGGAGAA TACAGTGATG | TTCCCTCTGCTGGCTCTTTC |
| *Ush1c* | CTTCTGCGAATCAAGAAGGAG | CCAGGGTGTAGTCCGTCAC |
| *Fst* | GAGGGAAAGTGTATCACAAAGTC | AGCTTCCTTCATGGCACACTC |
| *Otog* | TGACCTCTGCCAATCAGGAG | TTGCAGTGCACAGTGCATTG |
| *Tectb* | GCTGCTTTTGACCAGAGAGTG | CAAGACCACTTTGAACCGAAC |
| *Atp6v0a4* | GGCTCACGCTGAGCTGTC | TGGAATTCCACCCAATGCAG |
